# Traitement chirurgical à ciel ouvert des ruptures récentes du tendon calcanéen

**DOI:** 10.11604/pamj.2014.18.144.4335

**Published:** 2014-06-17

**Authors:** Hicham Mahdane, Abdesslam Khaissidi, Nasserdine Hammou, Mohammed Elidrissi, Shimi Mohamed, Abdelhalim Elibrahim, Abdelmajid Elmrini

**Affiliations:** 1 Service de Chirurgie Ostéoarticulaire B4, CHU Hassan II, Fes, Maroc

**Keywords:** Tendon calcanéen, rupture, chirurgie à ciel ouvert, Achilles tendon, rupture, open surgery

## Abstract

Les ruptures du tendon calcanéen quelles soient sous cutanées ou par section direct sont de plus en plus fréquentes. Plusieurs techniques chirurgicales, qu'elles soient à ciel ouvert ou percutanées ont été mises au point pour traitée ce type de lésion. À travers une étude rétrospective descriptive sur la prise en charge chirurgicale des ruptures de tendon calcanéen fraiches. Nous avons analysé les résultats fonctionnels de 38 patients, tous opéré par une chirurgie à ciel ouvert. Les points forts de notre étude étaient l'absence de rupture itérative, principale complication du traitement chirurgicale des ruptures du tendon calcanéen, un faible taux de complications liées à la chirurgie à ciel ouvert, ainsi que les résultats fonctionnels satisfaisants.

## Introduction

Les ruptures du tendon calcanéen quelles soient sous cutanées ou par section direct sont de plus en plus fréquentes. Le traitement chirurgical de ces ruptures vise à restituer l'anatomie et la continuité du tendon calcanéen et de l'aponévrose plantaire, afin de permettre une reprise des activités ultérieures. Plusieurs techniques chirurgicales ont été mises au point, à ciel ouvert ou percutanées. Dans notre étude nous avons utilisé une chirurgie à ciel ouvert, qui nous a permis une réparation non seulement anatomique, mais aussi solide du tendon calcanéen, avec des résultats très satisfaisants.

## Méthodes

Il s'agissait d'une étude rétrospective descriptive sur la prise en charge chirurgicale des ruptures de tendon calcanéen fraiches au service de chirurgie ostéoarticulaire B4 de janviers 2009 à Décembre 2013. Tous les patients opérés dans cette période ont été inclus dans l’étude, soit 38 patients. Ont été incus tous les patients traités chirurgicalement pour rupture du tendon calcanéen, qu'il s'agissait d'une rupture sous cutané ou d'une plaie ouverte. Il s'agissait de 19 ruptures sous cutanées survenues au cours d'un accident de sport (17 hommes et 2 femmes), avec 12 sportifs professionnelle, et 7 sportifs de loisir. Dans 19 cas les étiologies étaient variées (agression par arme blanche dans 13 cas, traumatisme direct dans 6 cas), tous des hommes. Nous avons revu les patients avec un recul moyen de 18 mois, le coté droit était le plus atteints (24 cas soit 63,15% des cas). L’âge moyen de nos patients était de 33,5 ans avec des extrêmes allant de 16 à 59 ans. L'anamnèse n'a pas révélé d'antécédents pathologiques chez nos patients, ni de tendinopathies chroniques du tendon calcanéen, et l'absence de notion de prise médicamenteuse (corticoïdes ou quinolone).

**Figure 1:**
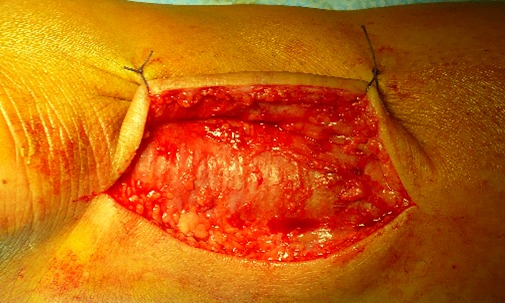
Abord para-achilléen médial, montrant la gaine tendineuse

**Figure 2:**
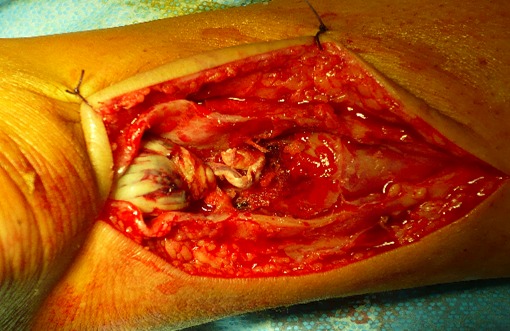
Ouverture longitudinale de la gaine tendineuse qui est disséquée

**Figure 3:**
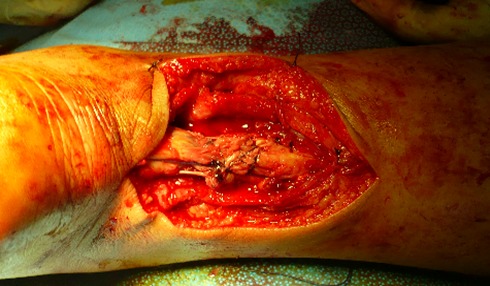
Suture tendineuse réalisée par des points de Kessler avec du fils à résorption lente, renforcée par un surjet

**Figure 4:**
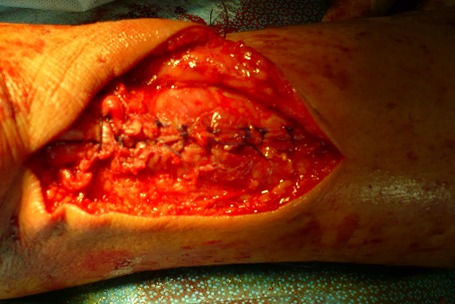
Fermeture de la gaine tendineuse, permettant ainsi un plan de glissement pour le tendon achilléen et évitant les adhérences

Dans notre étude, l’étiologie dans le groupe présentant une rupture sous cutanée était dominé par des sports d'impulsion (football dans 16 cas, puis le basketball dans 3 cas). Tous nos patients ont bénéficié d'un bilan radiologique standards qui était normal dans tous les cas, une échographie n'a été réalisé ont aucun cas, vue que nous jugeons que la clinique est suffisante pour établir le diagnostique. Tous nos patients ont été opérés par une technique à ciel ouvert dans un délai moyen de 1,5 jour (0-7 jours). Le patient était installé en décubitus ventral, avec garrot pneumatique à la racine de la cuisse. La voie d'abord était latéro-achilléenne médiale (Figure 1). La gaine tendineuse a été disséquée (Figure 2). La suture était réalisée par des points de Kessler avec du fils à résorption lente, renforcer par un surjet (Figure 3). La gaine tendineuse était ensuite refermée (Figure 4), ainsi que la peau, tout en faisant une suture étanche mais non ischémiante [1]. Une plastie par le plantaire grêle selon la technique de Chigo a été réalisée d'emblé chez 9 patients. Le protocole postopératoire était unique, il consistait en la mise en place d'une botte en équin pendant trois semaines puis d'une botte à 90’ pendant trois semaines et enfin à l'utilisation d'une talonnette de compensation pendant deux mois.

Les résultats fonctionnels ont été évalués objectivement par la mesure des amplitudes articulaire de la tibio-talienne, l'amyotrophie du segment jambier a été mesurée au niveau de sa plus grande circonférence, alors que la force musculaire a été évaluée par la possibilité d'appui sur la pointe du pied. Une évaluation fonctionnelle subjective, était basée sur la disparition de la douleur, ainsi que la reprise des activités sportives et du travail. Nous avons côté comme: **Excellents**: les tendons oubliés; **Bons**: discrets séquelles (gène au chaussage, appréhension lors des activités sportives; **Mauvais**: gène dans la vie courante.

## Résultats

Le recul moyen dans notre étude était de 18 mois. Les complications répertoriées comprenaient: un seul cas de nécrose cutanée superficielle ayant nécessité une reprise chirurgicale avec greffe de peau fine, un cas d'algodystrophie, et deux cas d'infections superficielles (chez deux patients présentant une rupture du tendon calcanéen par plaie ouverte) avec une bonne évolution sous antibiothérapie. L'analyse objective des résultats a montré que la dorsi-flexion ainsi que la flexion plantaire était symétrique par rapport au coté opposé dans 35 patients (92% des cas), diminuer chez 3 patients (8% des cas). La marche sur la pointe des pieds était possible chez 37 cas et l'amyotrophie était notée chez tous nos patients avec une moyenne de 15 mm, mais sans retentissement fonctionnel avec une récupération totale après rééducation. Le résultat subjectif a été jugé excellent chez 32 cas (84,3%) des cas, bon chez 5 cas (13,1%), et mauvais chez un seul patient (2,6%) ayant eu comme complication une algodystrophie. La durée moyenne d'arrêt de travail était de 75 jours. Le délai de reprise sportive était en moyenne de 190, et aucun sportif n'a arrêté sa pratique sportive, et seulement 79% des sportifs ont pu reprendre à leur niveau de pratique antérieur.

## Discussion

Notre série intéresse une population relativement jeune (âge moyen de 33,5 ans, et plus de 90% entre 16 et 40 ans) et comme la littérature la bien précisée nous retrouvons nous aussi une prédominance masculine. Dans le groupe des patients présentant une rupture sous cutanée du tendon d'Achille les accidents de sports étaient incriminés dans 100% des cas, nous retrouvons aussi un facteur de risque mécanique (surmenage sportif, sport occasionnel excessif) dans plus de 80% des cas. Par ailleurs nous n'avons pas trouvez de notion de tendinopathie achilléenne préexistante chez aucun de nos patients, un facteur retrouvez à des fréquences variable dans la littérature de 4% à 36% [2–4].

Avec un recul moyen de 18 mois, le résultat subjectif a été jugé excellent et bon chez 97,4%. Nous avons eu une amyotrophie inférieure à 2 cm (15 mm en moyenne) chez tous nos patients, retrouvée également dans les autres séries [5–8]. L'importance de l'amyotrophie variait en fonction de l'immobilisation: Häggmark et Eriksson [9] notaient une amyotrophie supérieure à 10% après six semaines d'immobilisation; au contraire après chirurgie et mobilisation précoce l'amyotrophie était inférieure à 2 cm dans les séries de Carter et al. Mais McComis et al [10] ont observé que l'amyotrophie n’était pas un critère très fiable, et que la technique opératoire n'a pas d'impact sur la récupération des amplitudes articulaires, seul la reprise précoce de l'appui et de la rééducation est gage de récupération des amplitudes articulaires comme Ozkaya et al [11] l'ont mis en évidence dans leur étude.

Les amplitudes articulaires des flexions dorsale et plantaire étaient grossièrement symétriques sauf pour trois patients chez qui on a noté une diminution de la flexion dorsale. Ce résultat est identique à celui rapporté par Lazrak [12], Delponte et al [6], Laffenetre et al [13], Lecestre et al [5]. La force musculaire du triceps sural a été évaluée par l'appui monopodal: celle-ci était similaire au côté sain sauf chez un patient, ce qui concorde avec les résultats de Kouvalchouk qui a noté 100% de symétrie par rapport au côté sain, mais de façon moindre pour d'autres: 31% pour Merti et al, et 73,7% pour Lecestre et al. Le retour à la vie active après chirurgie était un paramètre important. Dans notre étude il était en moyenne de 75 jours tout comme Cetti et al [14] ou encore Rouvillain et al [15].

Dans la littérature, la reprise sportive s'effectue entre 154 et 273 jours quelle que soit la prise en charge thérapeutique [11–16]. Au sein de notre population (patients sportifs, 19 cas), la reprise moyenne s'effectuait à 190 jours (120-240). Les complications liées à la chirurgie ont été classiquement rapportées dans la littérature [17], la plus redoutée étant la rupture itérative. Celle-ci surviendrait principalement durant les deux à trois premières semaines suivant l'opération et lors de la reprise de l'appui. Dans notre étude, aucun patient n'a présenté de reruptures. Ces résultats sont en accord avec la littérature [18–21]. Par ailleurs, nous avons retrouvé 10,5% de complications liées à la chirurgie, à type d'une nécrose cutanée superficielle chez un seul patient, deux cas d'infections superficielles du site opératoire, et une algodystrophie. Le faible taux de complications cutanées était probablement lié au suivi strict en postopératoire.

Les points forts de notre étude étaient l'absence de rupture itérative, principale complication du traitement chirurgicale des ruptures du tendon calcanéen, un faible taux de complications liées à la chirurgie à ciel ouvert, ainsi que les résultats fonctionnels satisfaisants.

## Conclusion

La chirurgie à ciel ouvert des ruptures du tendon calcanéen ne nécessitant pas une instrumentation spéciale, mais doit être réalisée avec une certaine rigueur pour éviter certaines complications, surtout cutanées. Dans notre étude, la qualité des résultats obtenus était très satisfaisante, aussi bien sur le plan anatomique, biomécanique que fonctionnel, ceci nous a permis d'adopter cette technique chirurgicale.
